# A review on extraction, composition, structure, and biological activities of polysaccharides from different parts of *Nelumbo nucifera*

**DOI:** 10.1002/fsn3.3376

**Published:** 2023-04-19

**Authors:** Mohib Ullah Kakar, Hammad Karim, Ghazala Shabir, Imran Iqbal, Muhammad Akram, Sajjad Ahmad, Muhammad Shafi, Pari Gul, Sania Riaz, Rizwan‐ur‐ Rehman, Hamid Salari

**Affiliations:** ^1^ Faculty of Marine Sciences Lasbela University of Agriculture, Water and Marine Sciences (LUAWMS) Uthal Balochistan Pakistan; ^2^ Sheikh Zayed Medical College Rahim Yar Khan Punjab Pakistan; ^3^ Fatima Jinnah Medical College Lahore Pakistan; ^4^ Department of Information and Computational Sciences School of Mathematical Sciences and LMAM Peking University Beijing China; ^5^ Department of Life Sciences, School of Science University of Management and Technology (UMT) Lahore Pakistan; ^6^ Faculty of Veterinary and Animal Sciences Lasbela University of Agriculture, Water and Marine Sciences (LUAWMS) Uthal Balochistan Pakistan; ^7^ Institute of Biochemistry University of Balochistan Quetta Pakistan; ^8^ Department of Bioinformatics and Biosciences Capital University of Science and Technology Islamabad Pakistan; ^9^ Department of Horticulture, Faculty of Agriculture Kabul University Kabul Afghanistan

**Keywords:** biological activities, lotus plant, nutraceutical potential, pharmaceutical potential, polysaccharide's extraction

## Abstract

*Nelumbo nucifera* (lotus plant) is an important member of the Nelumbonaceae family. This review summarizes the studies conducted on it since the past 15 years to provide an understanding on future areas of focus. Different parts of this plant, that is, leaves, roots, and seeds, have been used as food and for the treatment of various diseases. Polysaccharides have been extracted from different parts using different methods. The manuscript reviews the methods of extraction of polysaccharides used for leaves, roots, and seeds, along with their yield. Some methods can provide better yield while some provide better biological activity with low yield. The composition and structure of extracted polysaccharides have been determined in some studies. Although monosaccharide composition has been determined in various studies, too little information about the structure of polysaccharides from *N. nucifera* is available in the current literature. Different useful biological activities have been explored using in vivo and in vitro methods, which include antioxidant, antidiabetic, antitumor, anti‐osteoporotic, immunomodulatory, and prebiotic activities. Antitumor activity from polysaccharides of lotus leaves is yet to be explored, besides lotus root has been underexplored as compared to other parts (leaves and seeds) according to our literature survey. Studies dedicated to the successful use of combination of extraction methods can be conducted in future. The plant provides a therapeutic as well as nutraceutical potential; however, antimicrobial activity and synergistic relationships of polysaccharides from different parts of the plant need further exploration.

## Graphical abstract

*Nelumbo nucifera* is an important plant that has been used for therapeutic purposes and is an important part of human food. Leaves, roots, and seeds of this plant have been explored for extracting polysaccharides using different techniques and, in some studies, monosaccharide composition and structure have been determined. Several useful biological activities have been explored that have been discussed separately for each part of the plant in this review. These show a significant potential for this plant to be used for pharmaceutical as well as nutraceutical purposes. Antimicrobial potential has not been explored yet to the best of our knowledge from polysaccharides of this plant. Synergistic relationship of polysaccharides of this plant for therapeutic purposes remains to be studied, besides the determination of structure of polysaccharides.

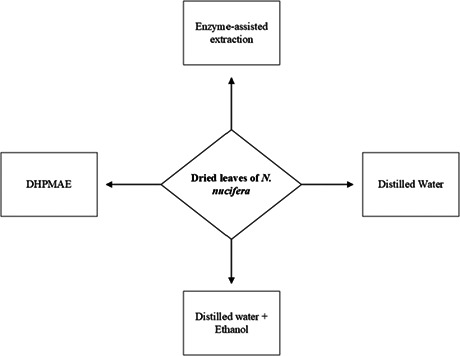

## 1 INTRODUCTION

*Nelumbo nucifera* also called lotus is an aquatic plant that belongs to Nelumbonaceae family. This plant possesses a characteristic fragrance and is also considered an ornamental plant. In several regions, it has different common names including sacred lotus, Chinese water lily, and Indian lotus. In almost all parts of Asia, it is used as a functional food, common vegetable, and herbal medicine for the past 2000 years (Guo, 2009). Nelumbonaceae family has only one genus which is further classified into two species, *N. lutea* and *N. nucifera* (Ming et al., 2013). Among the two, *N. nucifera* is mainly distributed throughout Asia and Northern Australia while *N. lutea* is found in the eastern and southern parts of North America (Yang et al., 2012). Geographically, this plant is widely distributed across China and is an important member of traditional Chinese medicine (TCM) with proven therapeutic effects through research.

*Nelumbo nucifera* is found largely in middle and lower regions near the Yangtze River in China (Guo, 2009). In TCM, *N. nucifera* seeds are used for the treatment of cancer, inflammation of tissues, as antiemetic, diuretic for children, and thermoregulator for the reduction of body temperature (Liu et al., 2004; Tang & Eisenbrand, 2013), as well as for the treatment of skin disorders (Mukherjee et al., 2010). Studies have been conducted for the isolation of bioactive compounds from this plant. Different parts of this plant, that is, leaves, seeds, flowers, and rhizomes have been used for the isolation of glycosides, flavonoids, triterpenoids, alkaloids, as well as vitamins. The bioactive compounds have been shown to exhibit therapeutic potential, hepatoprotective, and antioxidant activities (Huang et al., 2010; Isabelle et al., 2010; Mukherjee et al., 2009; Yen et al., 2006).

Polysaccharides are essential biological macromolecules that consist of monosaccharide units and are joined together through glycosidic linkages (Wang et al., 2017). These macromolecules are found together in plants, animals, as well as microorganisms naturally. These macromolecules have attracted considerable attention from researchers due to their low toxicity, biocompatibility, as well as numerous health benefits (Di et al., 2017). Polysaccharides derived from plants have shown certain potential activities. These polysaccharides have been reported to exhibit hypoglycemic, hypolipidemic, immunoregulatory, anticancer, antioxidant, and neuroprotective activities (Gao et al., 2018; Ho et al., 2016; Liu et al., 2015; Shin et al., 2016). Immunomodulation is one of the most important activities among them (Ferreira et al., 2015). Several reports have provided experimental evidence proving these biological macromolecules as important immune system regulators (Kim et al., 2016; Lee et al., 2013; Nie et al., 2018; Wang et al., 2017; Wu et al., 2017).

Specific activities demonstrated by polysaccharides are related to their monosaccharide composition, structure, glycosidic linkages, functional groups, and molecular weight (Ferreira et al., 2015). Several studies have demonstrated that immunostimulatory polysaccharides can activate dendritic cells, macrophages, and lymphocytes (Zheng et al., 2017). Polysaccharides obtained from plants can strengthen both adaptive and innate immune responses by triggering different events at molecular and cellular levels (Ferreira et al., 2015; Liu et al., 2015; Wang et al., 2016).

Different parts of *Nelumbo nucifera* have been utilized for the extraction of polysaccharides, their characterization, and determination of structure. Several useful biological activities have been reported by macromolecules obtained from this plant. Previously we have summarized the extraction, structure, composition, and biological activities of different compounds from different plants (Kakar et al., 2019, 2022; Kakar, Kakar, et al., 2020; Kakar, Naveed, et al., 2020). This review has been designed for summarizing the recent research conducted for the extraction of polysaccharides as well as their useful biological activities from different parts separately and an overview of the structure and composition of isolated polysaccharides has been provided. This study will give a better insight for future research on macromolecules from different parts of *N. nucifera* by identifying the areas which need further exploration.

## 2 EXTRACTION OF POLYSACCHARIDES FROM *NELUMBO NUCIFERA*

For studying polysaccharides from natural resources, extraction is the most important foremost step. Different methods are used for the extraction of polysaccharides traditionally which include maceration, chemical extraction, hot water extraction, and acid hydrolysis. These techniques are quite effective; however, they also have several drawbacks which include the application of high temperatures, considerable processing time, and utilization of large amounts of solvent. This can result in the extraction of non‐active components as well which in turn interfere with the yield of active components (Pan et al., 2015). Several other methods have been applied for the extraction which includes ultrasonic extraction, the use of microwaves, as well as enzymatic digestion. These methods have helped in the development of faster and more effective protocols for the extraction of polysaccharides (Marić et al., 2018). Enzyme‐assisted extraction (EAE) is another method that has been used as an efficient method for the extraction of polysaccharides as an environment‐friendly method (Wang, Liu, et al., 2018). These different methods as mentioned above have been used separately as well as in combination for the extraction of polysaccharides from different parts of *N. nucifera*. The application of different extraction methods as reported by various scientists is discussed for different parts of *N. nucifera* below.

### 2.1 Extraction of polysaccharides from leaves of *N. nucifera*

Leaves of *N. nucifera* have been an important source of natural polysaccharides. These macromolecules have been explored for possessing several useful biological activities after extraction, characterization, and determination of monosaccharide composition, structure, and molecular weight. Different methods have been used by scientists for this extraction which are discussed in this section.

Enzyme‐assisted extraction and hot water extraction were used by Song et al. (2020). Both types of extractions were performed for the purpose of comparison and determining the immunostimulatory activities as well as their physicochemical characteristics. For EAE, four different commercial enzymes were used, that is, α‐amylase, cellulase, pectinase, and protease. One hundred gram of dried lotus leaves were taken for this purpose. These were crushed and dispersed in distilled water. Two liter of distilled water was used at a ratio of 1:20 w/v, and then pH was adjusted for all the enzymes. For cellulase, amylase, and pectinase, the pH was adjusted to 4.5–5.0, and for protease, it was adjusted to 7.0. Each enzyme was added (1% v/w) and extraction was performed for 48 h at 50°C. For inactivation of enzyme, the mixture was heated at 95°C for 20 min. The mixture was centrifuged at 6000 ***g*** and supernatant was collected which was filtered using Whatman filter papers. In this aqueous extract, cold ethanol was added at 75% v/v final concentration, and then this mixture was kept overnight at −20°C. The mixture was precipitated, dissolved in water, and dialyzed using a membrane having a 6000–8000 Da cut‐off value. The retentate contained a high‐molecular‐weight fraction which was then lyophilized and stored for further use. Each enzyme extraction was named separately as LLEP‐C (extraction by cellulase), LLEP‐A (extraction by α‐amylase), LLEP‐P (extraction by pectinase), and LLEP‐PR (extraction by protease). For hot water extraction, a previously defined protocol (Song et al., 2019) was used. Distilled water was used for this extraction where 20 volumes of water was used, and extraction was performed twice for 4 h each at 100°C. Ethanol was used to precipitate crude polysaccharides from the distilled water. The mixture was then dialyzed as mentioned above using 6000–8000 Da membrane, and high‐molecular‐weight fraction (retentate) was lyophilized which was called LLWP. It was observed that both these techniques did not differ much in terms of the yield of polysaccharides; however, a considerable difference in the composition, characteristics, and immunostimulatory activities was observed among both types of extracted polysaccharides. Among all of them, pectinase‐assisted extraction was concluded the best in terms of immunostimulatory activities as compared to other polysaccharides obtained in this study (Song et al., 2020). Hot water extraction has always been a cheap and easy method as compared to EAE; however, these studies reflected that the use of enzymes (although expensive and difficult) for extraction is a better method. The method may not have much effect on the yield of polysaccharides; however, the obtained polysaccharides are important in terms of useful biological activities performed by them.

In a study, size exclusion chromatography was used for the extraction of water‐soluble polysaccharides from lotus leaves as described by Song et al. (2019). Here, 1 kg of dried leaves of *N. nucifera* was crushed and dispersed in 20 L of distilled water. In this study, 1 kg of dried leaves were used, which is much more in quantity as compared to the above‐mentioned study, as size exclusion chromatography requires an increased amount of inoculum for the process of extraction. Then, extraction was performed twice under reflux conditions and each time for 4 h. This extract was centrifuged for 20 min at 6000 ***g***. Then, supernatant was used for the precipitation using cold ethanol (75% v/v) for final concentration at 4°C overnight. The resulting precipitate was dissolved in water and dialyzed against 6000–8000 Da membrane. The resulting retentate was freeze‐dried. It resulted in the crude polysaccharide extract called LLWP‐C with a yield of 1.18%. Further purification was performed using gel permeation chromatography and Sephadex G‐100 having an approximate range from 4000 to 100,000 Da. For this purpose, the dimensions of the column used were 2.5 cm i.d. × 90 cm (Kim et al., 2016; Park et al., 2017). For elution of molecules, 50 mM ammonium formate buffer (pH 5.5) was used (flow rate 0.6 mL/min). The elutes were collected as 6 mL fractions in different tubes. For the fractions, protein, uronic acid, and neutral sugar analysis were performed, and based on molecular weight, four final subfractions were obtained. Then, 6000–8000 Da membrane was used for the dialysis of each fraction. After the removal of buffers, the retentate was lyophilized for obtaining four polysaccharides (LLWP‐1, LLWP‐2, LLWP‐3, and LLWP‐4) in pure form. Further, molecular weight as well as homogeneity were determined using HPLC. Based on further analysis of yield, purity of content, the crude polysaccharide LLWP‐C, and its two fractions LLWP‐1 and LLWP‐3 were used for further study. It was concluded that LLWP‐C and LLWP‐3 were the most effective in showing immunostimulatory activities as determined by experiments on RAW264.7 macrophages (Song et al., 2019).

In another study, leaves from this plant were put in a dried form in a lab grinder and sieved from 200 mesh after crushing. Later, the samples were stored in polyethylene bags and were kept at 4°C. This powder was used for dynamic high‐pressure microfluidization‐assisted extraction (DHPMAE) after mixing with water. Different ratios of sample and water were mixed at a liquid/solid ratio of 10:1 to 50:1 v/w and it was maintained before DHPMAE at different pressures. The range of pressures used was 80, 110, 140, 170, and 200 MPa for 1, 2, and 3 passes. Then, leaching was done for extraction at different temperatures, that is, 30–70°C. Re‐extraction of the residue was also performed twice after filtration of the extract obtained from the above‐mentioned process. Further, the filtrate was combined as well as concentrated to a final 100 mL volume at 60°C and under reduced pressure. A volume of 400 mL of ethanol (100%) was used for precipitation of the filtrate at 4°C for 12 h before performing 20‐min centrifugation at 1792 *g*. This process of precipitation was repeated twice. The obtained precipitate was then washed by using anhydrous ethanol and acetone after dissolving in 50 mL water to obtain lotus leaves polysaccharide termed LLP. In this study, for leaching, a sample and water were mixed at a concentration of 35:1 liquid/solid ratio before leaching for 1.5 h at 75°C. Extraction was done twice before concentrating the sample. For estimation of carbohydrate content of the obtained LLP, phenol–sulfuric acid method was used according to previous reports (Dubois et al., 1956). Glucose was used as a standard and after performing all extractions in triplicate, the final yield of polysaccharide was measured according to the following formula:

$$Y\;\left(\%\right)=\frac{M_{\mathrm{LLP}}}{M_{\mathrm{DM}}}\times 100$$

*M*_LLP_ in the above equation represents extract of polysaccharide from lotus leaves and *M*_DM_ shows the amount of powder of leaves used for this purpose. It was reported that DHPMAE yielded 6.31% polysaccharides under optimal conditions as compared to 2.31% yield obtained from leaching (Zhang et al., 2015).

Hwang et al. (2020) investigated the potential of lotus leaf‐extracted polysaccharide (LLEP) for osteoporosis as a suitable nutraceutical or pharmaceutical candidate. Dried leaves from the lotus plant were dispersed after crushing them. Distilled water was used for the dispersal at a 1:20 w/v ratio of the solid and liquid, respectively. pH was adjusted (4.5–5.0) and then pectinase was added at a ratio of 1% v/w of the raw material. The process was performed for 48 h at 50°C for extraction. Later, pectinase enzyme was inactivated after heating the mixture to 95°C for 20 min. The mixture was then centrifuged at 6000 ***g***. The supernatant was separated from the pellet and then it was filtered using Whatman No. 2 filter papers. Later, ethanol was added at 75% (v/v) final concentration and this solution was kept overnight at −20°C. It resulted in the formation of a precipitate which was again dissolved in water and then dialyzed using ultrafiltration for further purification 6000–8000 Da cut‐off membrane Spectra/Por® (Spectrum Laboratories Inc.). Polysaccharide fraction having high molecular weight obtained from this step was lyophilized and used further for the experiments. The final yield of polysaccharide was calculated as 1.2%. In later experiments, the molecular weight, monosaccharide composition, and sugar content from the obtained polysaccharide were determined. Later, in vivo experiments exhibited potential of LLEP in treating osteoporosis and it was the first study that reported beneficial effects of LLEP for the treatment of osteoporosis (Hwang et al., 2020).

Extraction targeting selenium‐containing polysaccharide was done from the lotus leaves by Zeng et al. (2017). Five hundred grams of clean air‐dried lotus leaf were dissected into small pieces and refluxed using ethanol (95%). The process was repeated twice at 75°C for 2 h. The process removed small pigments and lipophilic material. The mixture was centrifuged at 4000 ***g*** for 10 min. Then, degreased residue was processed for extraction using distilled water (10 volumes). The process was done at 100°C thrice for 3 h before its filtration using gauze. A rotary evaporator was used to concentrate the supernatant at reduced pressure. The sample was mixed with 95% ethanol and kept at 4°C overnight for precipitation of polysaccharides. The sample was centrifuged, frozen, and thawed thrice before treating with Sevag reagent for removing proteins. Twenty‐five gram crude polysaccharide (LLCP) was obtained which was lyophilized. Distilled water was used to dissolve the obtained 25 g LLCP and it was then filtered through 0.45 μm Millipore filter. The obtained filtrate was further purified using diethylaminoethyl (DEAE)‐cellulose column and it resulted in eluting of the fractions with distilled water, which resulted in neutral fraction (LLCP‐W). The fractions were followed by 0.2, 0.4, and 0.8 M NaCl at 2 mL/min flow rate to obtain the other fractions that were anion charged, these included, LLCPA, LLCPB, and LLCPC. All the elutes were monitored using phenol–sulfuric acid method (Dubois et al., 1956). The largest polysaccharide‐containing fraction was further subjected to Sepharose CL‐6B column. The fractions were eluted by using 0.1 M NaCl at 4 mL/min. Finally, 3.4 g of purified polysaccharide having yellow–white appearance was used for further study (Zeng et al., 2017).

The above studies show use of different methods for extraction of polysaccharides from the leaves of *N. nucifera*. Leaves have always been a difficult part for extraction purposes because of their chlorophyll content but the methods applied are quite successful in extraction of polysaccharides. Besides the availability of simple and easy methods like hot water extraction to the more complex methods; a combination of extraction methods is always a better choice for obtaining proper yield of useful polysaccharides.

**FIGURE 1:**
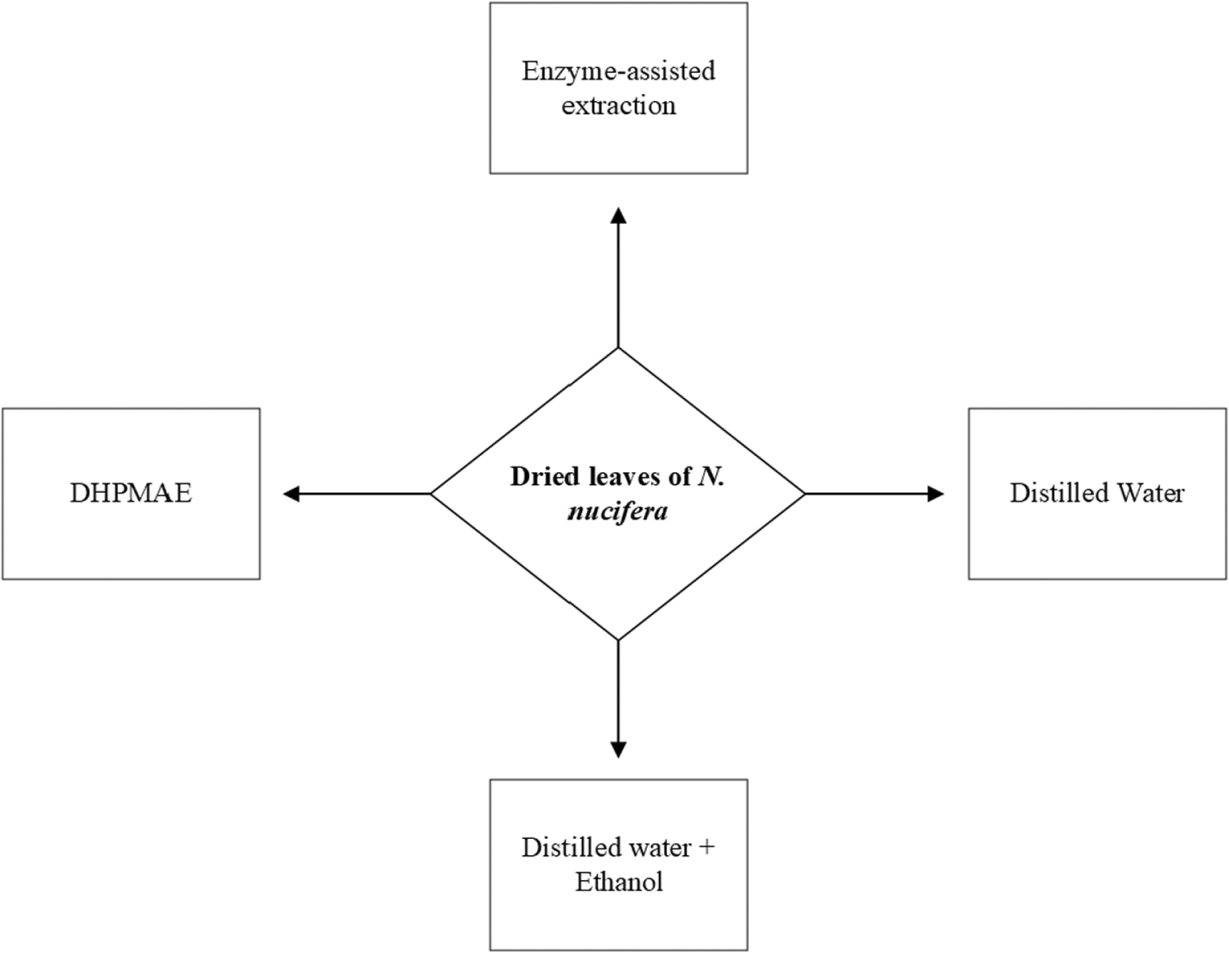
Schematic representation of methods of extraction used for polysaccharide extraction for *N. nucifera*.

A generalized overview of methods of extraction used from polysaccharide extraction for lotus leaves is given in Figure 1.

### 2.2 Extraction of polysaccharides from roots of *N. nucifera*

The roots of lotus plant are very important as 70% of them are used in the form of fresh food. Twenty percent are used to produce different products like vinegar, root powder, juices, etc. During this process, a large amount of root residues are produced (Hu et al., 2019). As most of the residue is wasted, it contributes to environmental pollution as well (Qiuliang et al., 2016). Healthcare industries can use agricultural residues and it can result in sustainable development. Lotus roots also contain polysaccharides as reported in previous studies (Yan et al., 2007). However, this resource has not been utilized to its potential due to lack of research on biological and chemical characteristics. Different scientists have extracted polysaccharides from lotus roots as well as lotus root residues. These techniques have been summarized here below:

Hu et al. (2019) used root residues of lotus plant for the extraction of polysaccharides. Distilled water was used to wash the root residues for the removal of starch. After washing, these were dried at 50°C in the oven. Fifty gram of this root residue was defatted using petroleum (500 mL) for 24 h and then deionized water (1.5 L) was used for the extraction at 90°C for 1 h. Supernatant obtained was concentrated under reduced pressure at 60°C. Proteins were removed using Sevag reagent after repeating the process three times. The obtained extract was precipitated using ethanol (threefold volume) by keeping the mixture at 4°C overnight. The obtained precipitate was centrifuged and anhydrous ethanol was used to wash it three times. It resulted in crude polysaccharide extract from the lotus root residue. Further, this extract was fractionated using DEAE Sepharose FF column and deionized water was used for eluting along with 0.1 M and then 0.3 M NaCl. Several fractions were obtained using deionized water and the active fractions were pooled for further purification. Sephadex G 100 column was used for further purification and eluted using deionized water. Pure polysaccharide was obtained at this step and the process was repeated several times for increasing the amount of polysaccharide obtained. The yield of the obtained polysaccharide was 1.23% (Hu et al., 2019).

In a previous study, lotus roots were dried, and 100 g were used for extraction. The sample was agitated for 6 h at 10°C using 1.4 L of distilled water. Low temperature was used because it is important for selectively extracting non‐starch polysaccharides. The mixture was shaken and then filtered before centrifugation at 1617 *g* for 5 min. Supernatant was used further. For precipitation of polysaccharides, absolute ethanol was used at a ratio of 4:1. The precipitate was recovered after centrifugation and then dried at 80°C using a stove. The extract obtained was weighed carefully and stored using silica gel for further study (Renato et al., 2007).

Wang, Yi, et al. (2018) analyzed 13 varieties of lotus roots for fingerprint profiling of polysaccharides. All the roots were cleaned and dissected before splitting them into three parts, that is, flesh, peel, and node. The materials were crushed using a food processor and then stored at −20°C for further processing. For extraction, 200 g of sample was used. It was homogenized in 2 L of distilled water at 16128 *g* for 5 min. It was then incubated for 3 h at 90°C before centrifugation at 3556 *g* for 10 min to separate the supernatant. Further, a rotary evaporator was used to concentrate the supernatant at 65°C and 0.1 MPa pressure to obtain a final volume of 200 mL. From this sample, proteins and starches were removed. 0.5 mL of alpha‐amylase reagent was used for the removal of starches, the mixture was incubated for 1 h in a water bath at 80°C along with the reagent. Proteins were removed by using the Sevag reagent. In this reagent, the volume of chloroform to *n*‐butyl alcohol was fourfold. For precipitation of polysaccharides, anhydrous ethanol was used and mixed with the sample at a ratio of 3:1 and kept overnight at 4°C. This precipitate was further centrifuged at 3556 ***g*** for 10 min and then it was washed with 75% ethanol. After washing it twice as mentioned, the precipitate was redissolved in 50 mL of distilled water and lyophilized. After lyophilization, the polysaccharides were weighed and kept at room temperature in a desiccator. The average yield of polysaccharides from the three mentioned parts was observed in the range 0.39–32 mg/g of fresh weight. These polysaccharides were further analyzed (Wang, Yi, et al., 2018).

Yi et al. (2019) extracted polysaccharides from lotus roots after dissecting and processing them in a food processor. The samples were homogenized at 7168 *g* for 5 min using distilled water (1:10 w/v). Resulting homogenate was stirred at 90°C for 3 h in a water bath at 2 *g*. This solution was centrifuged at 2268 *g* for 10 min to separate the extract. This extract was concentrated to one‐fourth of the starting volume at 65°C under vacuum. After concentration of solution, ethanol was used for the precipitation of starch from the extract. Thirty percent ethanol was used at 4°C for 3 h. Iodide/iodine reagent was used to confirm that starch was successfully removed. The non‐starch solution was centrifuged at 2268 *g* for 6 min for separation. Eighty percent ethanol was used for 6 h at 4°C for precipitating the polysaccharides. The solution was centrifuged at 2268 *g* for 6 min to collect the precipitate. This crude polysaccharide extract was lyophilized for further processes. 0.05 mol/L of phosphate buffer saline at pH 7.2 was used for dissolving the crude polysaccharide to obtain a final concentration of 5 mg/mL. The solution was filtered using 0.22 μm filter paper. Gel filtration chromatography was used for further purification using 8 mL of the sample and phosphate buffer saline as a mobile phase at 1.3 mL/min flow rate. The eluent was collected in tubes after every 6 min, and concentration was determined using phenol–sulfuric acid method (Dubois et al., 1956). The main fractions obtained were dialyzed using distilled water at 4°C for 72 h and lyophilized for further study (Yi et al., 2019).

In a recent study, Nawaz et al. (2021) extracted alkali‐soluble polysaccharides from cell wall of *N. nucifera* rhizomes named CWPs. For this extraction, the *N. nucifera* rhizome samples were collected, washed with distilled water, sliced, dried, grounded using pestle and mortar, and sieved before treating using a microwave oven at 200 W for 1, 2, 3, 4, and 5 min using a 30‐s interval after every treatment. Both microwave‐treated and native polysaccharides were kept in airtight bags for further use. One hundred gram of microwave‐treated and native polysaccharides from *N. nucifera* rhizome were treated for removal of cell wall. It means that the removal of cell was done by using the 10% potassium hydroxide (KOH), 100 mL in sodium borohydride (NaBH_4_), and 0.1% for 24 h. Further extraction was done using distilled water and a mixture of sodium acetate (0.05 M) and EDTA (0.05 M). Alkaline extract was reduced to 30 mL at 50°C in a water bath. The mixture was precipitated using 90 mL ethanol and the precipitate was centrifuged at 6000 ***g*** keeping the temperature at 4°C. The precipitate was washed using distilled water for the removal of excess salts. The resultant sample (CWPs) was freeze‐dried for further analysis. Extraction yield in alkali‐soluble CWPs in native and microwave‐treated NNRF ranged from 69.0 ± 1.00 to 54.33 ± 1.52% (Nawaz et al., 2021).

The methods used for extraction and their success have been mentioned including the procedures above; however, membrane extraction as a rapid separation method has not been used for polysaccharide extraction from the roots. The use of membranes with specific cut‐off values in combination with other methods can be a useful addition that may result in a better result in terms of obtaining useful polysaccharides.

**FIGURE 2:**
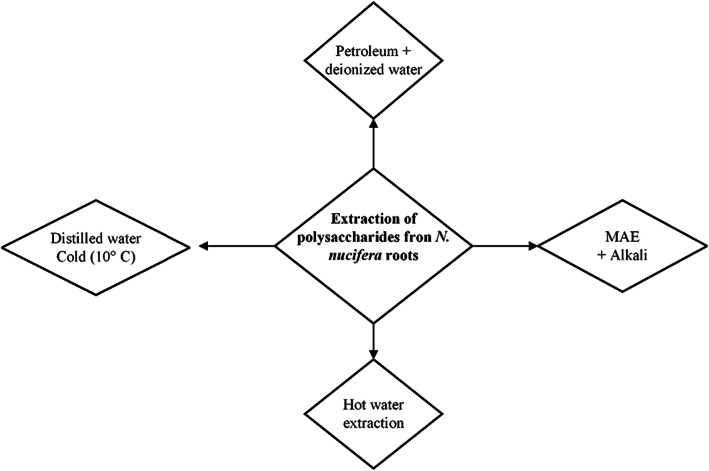
Schematic representation of methods of extraction for polysaccharides from *N. nucifera* roots.

A generalized overview of extraction methods from *N. nucifera* roots is given below (Figure 2):

### 2.3 Extraction of polysaccharides from seeds of *N. nucifera*

Seeds of *N. nucifera*, just like the roots and leaves, have been explored for the extraction, purification, characterization, and useful activities of polysaccharides possessed by them. Besides polysaccharides, other bioactive compounds are also present in lotus seeds which include saponins and phenolics (Ling et al., 2005). In this section, we will summarize the processes used for extraction of polysaccharides from seeds of *N. nucifera*.

Tian et al. (2012) reported the process of extraction of polysaccharides from lotus seeds in which seeds were harvested and processed by removing the seed coat, fruit coat having light purple color, spire, and the embryo root called plumula nelumbinis. The processed samples were air‐dried for 3 days at 45°C. The dried samples were processed using a mill and then sieved through a 60‐mesh sieve with 0.3 mm pore size. The obtained powder was kept in sealed plastic bags in a desiccator at room temperature. An ultrasound clearer instrument was used for the ultrasound‐assisted extraction of polysaccharides from dried seed powder. Ten gram powder was dissolved in 150 mL distilled water using beaker. The mixture was held in ultrasound clearer, and extraction was performed at different parameters. Ultrasound power was used from 360 to 440 W and temperature range 90–100°C for the time ranging from 6 to 10 min. The resulting solution was filtered for removal of debris and then concentrated using speed vacuum concentrator. Proteins were removed from this mixture using Sevag reagent (Sevag et al., 1938). The resulting solution was precipitated using 95% ethanol (3:1) after keeping the mixture at 4°C overnight. This solution was centrifuged at 2268 g for 20 min at 4°C to collect the precipitate. The obtained precipitate was further washed using distilled water and centrifuged. The resulting precipitate was freeze‐dried under vacuum and it yielded crude polysaccharide from the lotus seed called LSPS which was weighed carefully on analytical balance. The % yield of polysaccharides was calculated as:

$$\text{Yeild}\;\left(\%\right)=\frac{\text{Weight of dried crude extraction}}{\text{Weight of lotus seed powder}}\times 100$$

For optimization of parameters of ultrasound‐assisted extraction, a three‐level, Box–Behnken design (BBD) was used, and different variables were assessed in the light of % yield. It was concluded that 1.51% yield of crude polysaccharide was obtained under the optimized parameters, 406 W, 7.7 min, and 94°C. In the same study, hot water extraction (HWE) was also performed in a water bath. Distilled water was used in a ratio of 15:1 as compared to the sample at 100°C for 180 min for this purpose. Microwave‐assisted extraction for polysaccharides from lotus seeds was performed in an automated apparatus at 350 W and 80°C for 6 min using 15:1 solvent volume (Tian et al., 2012).

Lu et al. (2015) studied the effect of water‐soluble oligosaccharides on the growth of *B. adolescentis*, a useful bacterium under the conditions of gastric acid secretions. For this purpose, lotus seeds in frozen form having their cores removed were used. These were mixed with distilled water for 1 h at a ratio of 3:10 w/v and then homogenized using a high‐speed electric tissue homogenizer. Further α‐amylase was used for the removal of starch through hydrolysis at 65°C. Iodine was used to determine the products after digestion. For the removal of protein, papain was used to treat the lotus seed extract after cooling the extract to 55°C. The process continued until stable protein concentration was obtained. For the removal of monosaccharides, 1% yeast was added at 37°C at the start of fermentation. When the sugar content became stable, the process of fermentation was stopped. The resulting suspension was heated for 2 h at 90°C in autoclave. This process resulted in the inactivation of any enzyme present in the solution. The suspension was mixed with 95% ethanol. Five volumes of ethanol were used, and the mixture was kept at 4°C overnight for precipitation. The mixture was centrifuged at 3100 ***g*** for 20 min. It resulted in the removal of polysaccharides from the extracted sample. The supernatant obtained after the process was again subjected to removal of proteins. It was mixed with 5% trichloroacetic acid (TCA) using 1.5 volumes of TCA and then the mixture was kept at 4°C for 6 h. The solution was centrifuged again at 3100 ***g*** for 20 min and the presence of protein was determined by using Ninhydrin reagent test. The supernatant was collected which contained the crude lotus oligosaccharide (LOS). Further purification with the help of resin was also done eluted by using distilled water. Sugar content was determined by previously established phenol–sulfuric acid method (Dubois et al., 1956). The fractions were collected according to the peaks given by phenol–sulfuric acid method. Both crude and purified oligosaccharides, LOS and P‐LOS, respectively, were freeze‐dried for further study (Lu et al., 2015).

As oligosaccharides from lotus seeds are important compounds acting as prebiotics, extraction parameters for ultrasound microwave‐assisted extraction (UMAE) for obtaining a high yield of oligosaccharides were optimized through response surface methodology which included temperature, extraction time, ratio of solvent, etc. Lu et al. (2017). For this purpose, fresh quick frozen lotus seeds were used after thawing for 1 h followed by air drying. The seeds were dried at 50°C so that they retain only 7% of their original water content. Later, desiccated lotus seeds were crushed using a mill and sieved through 60 meshes having 0.3 mm pore size. This dried seed powder was mixed with deionized water at a ratio of 1:5 w/v. From this sample, starch was removed according to the procedure described by Guo et al. (2015). After removal of starch, the solution was added to 150 mL constant‐volume liquid–solid ratio, as applied in the response surface design. The resulting solution was transferred for ultrasonic microwave‐assisted extraction to a three‐vase glass‐type heterotypic in the instrument. Once the reaction was completed, vacuum aspiration was applied to the solution and the residue obtained was discarded. After the concentration of filtrate using speed vacuum, three volumes of ethanol (95%) were added, and the mixture was kept at 4°C overnight. The mixture was then centrifuged, supernatant separated, and freeze‐dried for further analysis. Hot water extraction and ultrasonic‐assisted extraction were also performed in this study according to the parameters determined by the response surface methodology (RSM). It was concluded that the optimum conditions for ultrasound microwave‐assisted extraction (UMAE) included a liquid–solid ratio of 10 g/mL, time 325 s, microwave power 250 W, and ultrasonic power 300.46 W. The yield of tetra‐, tri‐, and oligosaccharides in this method was predicted theoretically as 4.894%, 3.947%, and 3.016%. The study showed a method for increased yield with shortened extraction time (Lu et al., 2017).

Zheng, Wang, Zhuang, et al. (2016) used dried lotus seeds for the extraction of polysaccharides using hot water extraction. Seed powder was incubated using 10‐fold water at 100°C for 3 h. Vacuum filtration was used for further separation of supernatant and sediment. Rotary evaporator under vacuum was used at 55°C for concentrating the extract. Ethanol (95%) was used for the precipitation of polysaccharide. fourfold of ethanol was used, and mixture was kept at 4°C. The precipitate was separated and dissolved in deionized water. It was deproteinized using 10% plumbous acetate. The resulting solution was centrifuged, and the supernatant was separated. After concentration of supernatant, it was dialyzed at 4°C for 48 h. The resulting solution was freeze‐dried under vacuum and crude lotus seed polysaccharide (LSPS) was obtained for further use. A 0.95% yield of LSPS was recorded using hot water extraction (Zheng, Wang, Zhuang, et al., 2016).

Lotus plumule polysaccharides have been extracted in many studies which we will discuss here. Liao et al. (2011) reported the process of extraction in which lotus seeds were collected, air‐dried at 40°C, powdered, and then subjected to hot water extraction. Deionized water (5:1) was mixed with the sample and kept for 4 h at 100°C in a water bath. The extract was centrifuged for 30 min at 5000 ***g***, and supernatant was collected. It was mixed with 95% ethanol and kept at 4°C overnight to precipitate the polysaccharide. This mixture was centrifuged at 5000 ***g*** for 30 min and pellet was collected having lotus plumule polysaccharide (LPPS) which was lyophilized and stored at −30°C for further use (Liao et al., 2011). The same process of extraction as described in this study was used in another study conducted by Liao and Lin (2011). The yield of polysaccharide obtained in this study was reported as 3.86 ± 1.47%. Similarly, Liao and Lin (2013a) also used the same method of extraction for LPPS extraction which was later purified into two fractions called F1 and F2 using Sepharose 6B gel filtration (Liao & Lin, 2013a).

In a recent study, Le et al. (2021) used lotus seeds that were air‐dried at 65°C using intermittent hot air at a speed of 2.0 m/s. After 72 h of drying, plumule was separated, crushed, and powdered before pretreating it at 50°C using anhydrous ethanol. This process was done to separate the pigments from the sample. Two hundred gram of this pretreated sample was mixed with 10 volumes of distilled water at 92°C for 5 h. Extract from this mixture was collected after centrifugation at 900 ***g*** for 15 min. The pellet was again mixed with distilled water and centrifugation was repeated as in the previous step for collection of supernatants. Both supernatants were combined. These were concentrated at 55°C, under reduced pressure, and then precipitated using ethanol. Three volumes of 95% ethanol were used, and the mixture was kept at 4°C for 24 h. The precipitate was collected after centrifugation of the mixture at 900 ***g*** for 15 min and proteins were removed from it using Sevag reagent. This resulted in the extraction of *N. nucifera* plumule polysaccharide which was termed NNP. This was further purified using gel permeation chromatography into two fractions, NNP‐1 and NNP‐2, which were further analyzed, and NNP‐2 was concluded as the main fraction obtained in this study (Le et al., 2021).

Among the extraction methods of polysaccharides from the seeds, a combination of methods for extraction from the different parts of the seed like seed coat, whole seed, etc. can be applied and compared. It may give better results in terms of polysaccharide yield and its therapeutic utility. Membrane extraction process has not been used for polysaccharide research from the seeds, which can be applied in future research.

**FIGURE 3:**
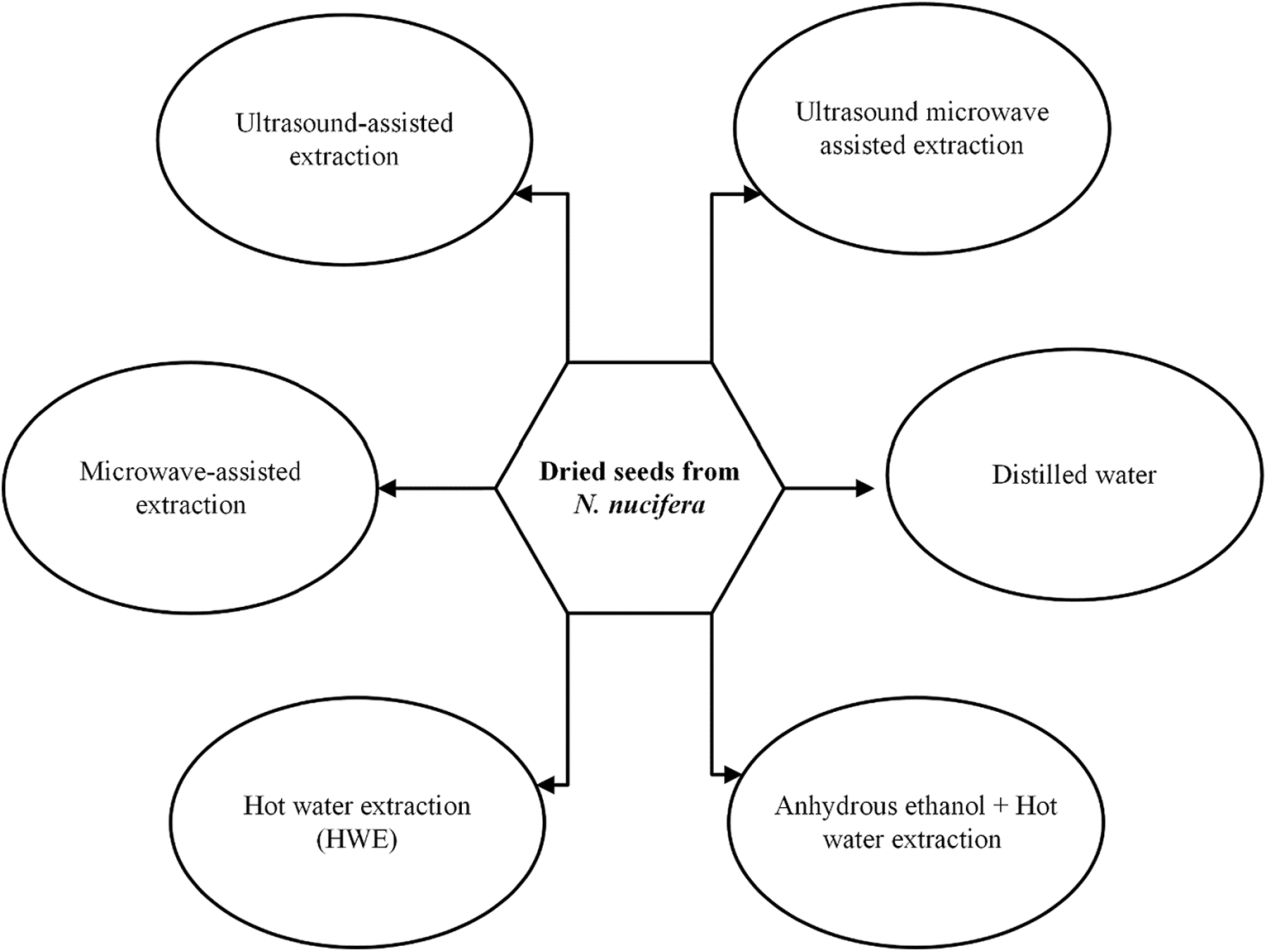
Schematic representation of methods of extraction used from polysaccharide extraction from *N. nucifera* roots.

An overview of methods of extraction used for polysaccharide extraction from lotus seeds is given in Figure 3.

Table 1 given below shows a general summary of the methods of extraction for polysaccharides extraction from *N. nucifera*. Different parameters used for extraction, including time, temperature, etc., are also included. The % age yield obtained after using each type of method is also shown in the table.

**TABLE 1:** A summary of methods extraction used for extracting polysaccharides from *N. nucifera.*

Name	Method of extraction	Time (min)	Temperature, °C	Water/Material ratio (g/mL)	Power (V)/Pressure (MPa)	Solvent	Yield (%)	References
LLEP‐A	Enzyme‐assisted extraction	48 h	50	1:20	–	Distilled water	0.97	Song et al. (2020)
LLEP‐C							1.17	
LLEP‐P							1.11	
LLEP‐PR							1.93	
LLPs	DHPM‐assisted extraction	50	76	35:1	80 MPa	Distilled water	6.31	Zhang et al. (2015)
LSPS‐1	Ultrasound‐assisted extraction (RSM)	7.7	94	1.15	406	–	1.51	Tian et al. (2012)
LPP	Ultrasound‐assisted extraction (RSM)	32.87	92.86	28.32	–	Distilled water	13.92	Zhang et al. (2017)
Oligosaccharides	Ultrasound microwave‐assisted extraction (UMAE), (RSM)	5.4	Room temp	1:10	300.46	Distilled water	3.01	Lu et al. (2017)
LPPS	Hot water extraction	240	100	1:10	–	Distilled water	–	Liao and Lin (2013a)
LLWP‐C	Hot water extraction	240	100	1:20	–	Distilled water	–	Song et al. (2019)

## 3 STRUCTURE OF POLYSACCHARIDES FROM *N. NUCIFERA*

The structure of polysaccharides is one of the most important factors that determine the useful biological activities of specific polysaccharide molecule. The composition of monosaccharides, molecular weight, and the position and sequence of glycosidic bonds are all important factors in determining the properties and structure of polysaccharides (Chen et al., 2013). In the case of polysaccharides from lotus plants, only a few studies have been reported in which structure has been elucidated. This aspect still remains underexplored. Only polysaccharides from lotus roots have been further studied for structure determination.

**FIGURE 4:**
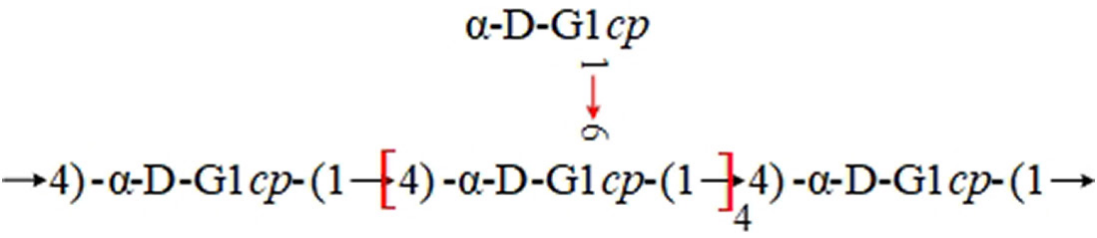
Structure of polysaccharide from lotus root as depicted by Hu et al. (2019)).

Yi et al. (2019) reported that polysaccharide isolated from lotus root (LRP) consisted of Glc‐(1→, →6)‐Glc(1→, →6)‐Gal‐(1→, →4,6)‐Gal‐(1 → and →3,6)‐Glc‐(1→), which were the major linkages. The main residue of LRPs determined in this study was (1 → 6)‐linked D‐glucopyranosyl, and the main repeating unit was 1,6‐linked glucose. From this study, it was concluded that the polysaccharides were mainly α‐(1 → 6)‐D‐heteroglucans. These were composed of Glc‐(1→, →6)‐Glc‐(1→, →6)‐Gal‐(1→, →4,6)‐Gal‐(1 → and →3,6)‐Glc‐(1→) and molar ratio was reported as 1.00:4.33:0.83:0.13:1.14 (Yi et al., 2019). In another study (Hu et al., 2019), the structure of polysaccharide isolated from lotus root residues was determined. It was determined to have α‐D‐(1 → 4)‐linked glucopyranosyl moieties. These had non‐reducing terminal α‐D‐Gl*cp* at O‐6 as branches every six residues. The structure is illustrated in Figure 4 below:

## 4 COMPOSITION OF POLYSACCHARIDES

Various studies have elucidated the composition of polysaccharides extracted and purified from different parts of *N. nucifera*. In this section, a summary of monosaccharide composition is presented in tabular form for simplicity (Table 2). As the molecular weight of polysaccharides and obtained yield are also important factors, reactive yields and their molecular weights are also summarized besides the biological activities reported from the extracted and purified polysaccharides.

**TABLE 2:** A summary of monosaccharide composition, percentage yield, molecular weight, origin, purity percentage with respect to polysaccharide composition, and biological activities.

Name	Origin	Molecular weight (kDa)	Monosaccharide composition	Composition (%age)	Biological activities	Yield (%)	References
LLEP‐A	Seoul, Korea	399	Fuc:Rha:Ara:Gal:Glc:Man:Xyl:GalA: GlcA 0.77:8.52:22.63:24.94:2.93:4.31:2.55:30.63:2.71	N.S 65.36 U.A 32 Pro 2.04	Immunostimulatory effect	0.97	Song et al. (2020)
LLEP‐C		150.8	Fuc:Rha:Ara:Gal:Glu:Man:Xyl:GalA: GlcA 0.77:8.21:22.89:26.18:4.02:2.82:3.14:29.66:2.31	N.S 66.9 U.A 31 Pro 1.32		1.17	
LLEP‐P		78.8	Fuc:Rha:Ara:Gal:Glu:Man:Xyl:GalA: GlcA 1.25:11.9:21.21:29.77:3.5:4.43:2.08:21.8:4.06	N.S 68.66 U.A 28.7 Pro 1.52		1.11	
LLEP‐PR		89.9	Fuc:Rha:Ara:Gal:Glc:Man:Xyl:GalA: GlcA 0.36:4.18:16.63:22.86:26.51:1.80:2.68:13.88:11.11	N.S 80.47 U.A 15.6 Pro 3.31		1.93	
LLWP		70.6	Fuc:Rha:Ara:Gal:Glc:Man:Xyl:GalA: GlcA 0.77:5.95:15.05:19.51:12.31:3.85:4.08:36.17:2.31	N.S 54.6 U.A 43.8 Pro 0.9		1.18	
WSF	Hubei Province, China			U.A			Liu et al. (2020)
Fresh			Man:Rha:Glc:Gal:Xyl:Ara:Fuc 1.0:2.7:32.4:11.5:0.2:4.1:0.1	48			
Distilled			Man:Rha:Glc:Gal:Xyl:Ara:Fuc 1.9:5.2:10.7:20.2:0.2:7.5:0.4	53			
Acetic acid			Man:Rha:Glc:Gal:Xyl:Ara:Fuc 1.8:5.3:8.9:21.5:0.2:7.1:0.3	55			
CSF							
Fresh			Man:Rha:Glc:Gal:Xyl:Ara:Fuc 0.3:1.2:63.5:4.6:0.2:3.0:0.3	27			
Distilled			Man:Rha:Glc:Gal:Xyl:Ara:Fuc 0.7:3.5:1.5:9.9:0.1:3.2:0.0	81			
Acetic acid			Man:Rha:Glc:Gal:Xyl:Ara:Fuc 0.3:3.2:5.1:8.7:0.0:2.7:0.1	83			
NSF							
Fresh			Man:Rha:Glc:Gal:Xyl:Ara:Fuc 0.1:2.0:5.7:6.5:0.0:1.9:0.0	83.8			
Distilled			Man:Rha:Glc:Gal:Xyl:Ara:Fuc 0.2:1.9:0.6:5.3:0.0:1.8:0.0	90.2			
Acetic acid			Man:Rha:Glc:Gal:Xyl:Ara:Fuc 0.2:1.9:1.5:6.9:0.0:1.9:0.0	87.5			
HF							
Fresh			Man:Rha:Glc:Gal:Xyl:Ara:Fuc 0.6:2.8:30.8:32.4:14.7:7.3:0.0	11.4			
Distilled			Man:Rha:Glc:Gal:Xyl:Ara:Fuc 0.6:3.1:19.9:30.1:22.8:8.1:0.0	15.2			
Acetic acid			Man:Rha:Glc:Gal:Xyl:Ara:Fuc 0.8:1.9:46.7:22.6:14.0:5.5:0.0	8.6			
LLWP‐C	–	–	Fuc:Rha:Ara:Gal:Glc:Man:Xyl:GalA:GlcA 0.77:5.95:15.05:19.51:12.31:3.85:4.08:36.17:2.31	N.S 46.1 U.A 52.3 Pro 0.27	Immunostimulatory effects	–	Song et al. (2019)
LLWP‐1		85.1	Fuc:Rha:Ara:Gal:Glc:Man:Xyl:GalA:GalC 0.34:6.98:24.76:27.96:5.98:1.01:3.94:26.37:2.66	N.S 52.8 U.A 46.3 Pro 0.10			
LLWP‐3		12.5	Fuc:Rha:Ara:Gal:Glc:Man:Xyl:GalA:GlcA 1.05:6.60:9.79:15.01:8.85:6.09:3.97:47.19:1.46	N.S 45.5 U.A 52.9 Pro 0.03			
LRP	Jiangsu Province, China	12.4	Glc only	CH_2_0 70.08 Pro 0.16 U.A 0.46	immunomodulatory activity	1.23	Hu et al. (2019)
SSPS	Jiangsu Province, China	–	–	CH_2_0 88.9 Pro 4.1	–	–	Liu et al. (2019)
LSPS	Fujian, China	–	–	–	Immunomodulatory, antitumor effect MFC, HuH‐7, H22	0.95	Zheng, Wang, Zhuang, et al. (2016)
LSPS	Fujian, China	–	–	–	Antitumor and immunomodulatory activities		Zheng, Wang, Lu, et al. (2016)
LRPs	Wuhan, China		–	–	Antioxidant, immunostimulatory activities anticancer effect SGC‐7901, HepG2		Yi et al. (2018)
LPPS	Tainan, Taiwan	391	Glc:Gal:Fru:Xyl:Fuc 25.7:10.5:22.0:33.4:8.1	CH_2_O 42.1 Pro 38.3 Phenolic 3.1	Anti‐inflammatory effects		Liao et al. (2011)
LPPS	Tainan, Taiwan	391	–	CH_2_O 70 Pro 30	Anti‐inflammatory effects		Liao and Lin (2012)
F‐1		2		CH_2_O 69.9 Pro 30.1			
F‐2		25.7		CH_2_O 96.5 Pro 3.5			
LLP‐D	–	550	Rha:Fuc:Ara:Xyl:Man:Glc:Gal 6.83:0.57:2.73:3.25:9.20:19.23:58.19	–	Antioxidant activity	6.31	Zhang et al. (2015)
LLP‐L		578	Rha:Fuc:Ara:Xyl:Man:Glc:Gal 5.03:7.04:19.39:2.21:6.07:22.82:37.45				
LPPS	Tainan, Taiwan	–	–	–	Antidiabetic activity	–	Liao and Lin (2013a)
LPPS	Tainan, Taiwan	–	–	–	Hepatoprotective, anti‐inflammatory, and antidiabetic effects	–	Liao and Lin (2011)
LPPS	Tainan, Taiwan	391		CH_2_O 70 Pro 30			
F‐1		2		CH_2_O 69.9 Pro 30.1			Liao and Lin (2013b)
F‐2		25.7		CH_2_O 96.5 Pro 3.5			
LSPS‐1	Fujian Province, China	4.48	Rha:Ara:Glc:Gal 7.13:4.81:13.28:1.00	CH_2_O 96.5 U.A 2.33 Sulphate 1.24	Antioxidant activity	1.51	Tian et al. (2012)
LPP	Jinan, China	–	–	–	Antioxidant activity	13.92	Zhang et al. (2017)
LLP	Zhejiang, China	–	–	CH_2_O 93.4 20.23 μg/g of Se	Antidiabetic activity	–	Zeng et al. (2017)
Oligosaccharides	Fujian, China	–	Glc:XOS:FOS:LOS:P‐LOS	–	Probiotic activity	–	Liu et al. (2015)
Oligosaccharides	Fujian, China	–	–	–	–	3.01	Lu et al. (2017)
LRPs	Wuhan, China	5.94	Man:Rha:GlcA:GalA:Glc:Gal:Ara 0.19:0.14:0.08:0.17:6.49:1.00:0.16	–	–	–	Wang, Yi, et al. (2018)
LRPs	Wuhan, China	1.33–5.3	Man:Rha:GalA:Glc:Gal:Ara 0.19:0.14:0.17:6.49:1.00:0.16	CH_2_O 94.8 Pro 3.54	Antioxidant, Anti‐tumor, SGC7901, and HepG2 cancer cells	–	Yi et al. (2019)
LLEP	Seoul, Korea	76.8	Rha:Ara:Gal:Man:Glc:GalA 11.9%:21.2%:29.8%:4.4%:3.5%:21.8%	–	Anti‐osteoporotic effects	1.2	Hwang et al. (2020)
NNP‐2		110.47	Xyl:Glc:Fru:Gal:Fuc 33.4:25.7:22.0:10.5:8.1		Prebiotic activity/anti‐diabetic	83.43	Le et al. (2021)

Abbreviations: Ara, arabinose; CH_2_O, carbohydrate; CSF, chelate‐soluble fraction; DHPM, dynamic high‐pressure micro‐fluidization; FOS, fructo‐oligosaccharides; Fru, fructose; Fuc, fucose; Gal, galactose; GalA, galacturonic acid; Glc, glucose; GlcA, glucuronic acid; H22, mouse hepatocarcinoma cells; HepG2, human liver cancer cell line; HF, hemicellulose fraction; HuH‐7, human liver cancer cells; LLEP‐A, lotus leaf polysaccharides were extracted by enzyme‐assisted extraction by using α‐amylase; LLEP‐C, lotus leaf polysaccharides were extracted by enzyme‐assisted extraction by using cellulose; LLEP‐P, lotus leaf polysaccharides were extracted by enzyme‐assisted extraction by using pectinase; LLEP‐PR, lotus leaf polysaccharides were extracted by enzyme‐assisted extraction by using protease; LLPs, lotus leaves polysaccharides; LLWP, lotus leaf polysaccharides were extracted by hot water extraction; LOS, lotus seed oligosaccharides; LPP, lotus plumule polysaccharides; Man, manose; MFC, mouse gastric cancer cells; N.S, neutral sugar; NNP, *N. nucifera* polysaccharide; P‐LOS, purified lotus seed oligosaccharides; Pro, protein; Rha, rhamanose; Rib, ribose; RSM, response surface methodology; SGC‐7901, human gastric cancer cell line; SSPS, soybean‐soluble polysaccharide; U.A, uronic acid; WSF, water‐soluble fraction; XOS, Xylo‐oligosaccharides; Xyl, xylose.

## 5 BIOLOGICAL ACTIVITIES OF POLYSACCHARIDES ISOLATED FROM DIFFERENT PARTS OF *N. NUCIFERA*

As mentioned in the preceding sections, different parts of *N. nucifera* have been used for the treatment of various diseases in TCM. Over the years, scientific evidence of useful biological activities has been explored in different studies by the application of polysaccharides in vivo and in vitro. This part of our review focuses on the reports showing useful biological activities of polysaccharides extracted from parts of lotus plants. Each part has been discussed separately for a better overview of the reported biological activities.

### 5.1 Biological activities from polysaccharides of lotus leaves

Several useful biological activities have been reported from the polysaccharides extracted from lotus leaves which are summarized below.

#### 5.1.1 Antidiabetic activity

The effect of Se‐containing lotus leaf polysaccharide Se‐LLP was investigated on gestational diabetes mellitus (GDM) in mice by Zeng et al. (2017). Wistar rats having body weights from 202 to 248 g were used in this study where pregnant females after conception were injected with 2% streptozotocin subcutaneously. The dose was administered at 40 mg/kg body weight for inducing GDM. The rats were administered lotus leaf polysaccharide (LLP) that contained 20 μg/g organic Se at 50 mg/kg body weight and 100 mg/kg body weight in separate groups over 2 weeks period. The final analysis of rats after C‐section, collection of fetus and placenta was conducted for different parameters to establish the possible therapeutic potential of administered LLP. It was reported that LLP administration resulted in significant relief of diabetic symptoms, increased glycogen content in the liver, stable body weight as compared to weight loss occurring in diabetes, lower fasting blood glucose, and less fasting blood insulin levels. Moreover, 2 weeks of LLP treatment also helped in treating liver abnormalities occurring in the case of GDM, which was evident by the low level of total cholesterol, total triglyceride, and low‐density lipoprotein, and increased level of high‐density lipoprotein. Besides an improved antioxidant activity among the liver enzymes was also observed, which provided an evidence for potential future therapy against diabetes mellitus as well as its complications (Zeng et al., 2017).

#### 5.1.2 Immunostimulatory activity

Polysaccharides extracted from lotus leaf have been reported to exhibit immunostimulatory effects in vitro when applied to RAW264.7 cells; moreover, difference in the efficiency of polysaccharides obtained from enzymatic extraction and hot water extraction was also compared (Song et al., 2020). Among the different parameters (concentrations of 3, 10, and 30 μg/mL), phagocytic activity of macrophages was reported to be promoted using all the polysaccharides except for the one extracted using protease. However, polysaccharide molecules extracted from enzymatic extraction showed better efficiency in promoting phagocytic activity of macrophages as compared to those extracted using hot water extraction method. Besides, LLEP‐P, that is, lotus leaf extracted polysaccharides using pectinase was found to be the most potent enhancer of phagocytic activity of macrophages. Increased production of NO and cytokines (TNF‐α, IL‐1β, and IL‐6) was observed using LLEP‐P in a dose‐dependent manner as compared to other extracted polysaccharides. Other enzymes used for extraction were cellulase (fraction LLEP‐C), α‐amylase (fraction LLEP‐A), and protease (fraction LLEP‐PR), besides hot water extraction (fraction LLWP). The highest activity was shown by fraction extracted using pectinase, followed by cellulase, α‐amylase, hot water, and protease. The composition of lotus leaf polysaccharides was observed as rhamnose, arabinose, galacturonic acid, and galactose. The compositions and molecular weights differed among different extraction processes. However, enzymatic‐assisted extraction using pectinase was concluded as the best method for enhanced immunostimulatory activity, and the extracted polysaccharide using protease was reported as the worst method for this purpose (Song et al., 2020).

In another study conducted by Song et al. (2019), crude polysaccharide was extracted from the lotus leaf (LLWP‐C) which was further purified and yielded four fractions LLWP‐1, LLWP‐2, LLWP‐3, and LLWP‐4. All the fractions were homogenous and out of the four, two fractions LLWP‐1 and LLWP‐3 having 19.9% and 21.3% yield were further applied on RAW264.7 macrophages for studying immunostimulatory effects. LLWP‐1 had a molecular weight of 8.51 × 10^4^ and LLWP‐3 1.25 × 10^3^ Da. Different concentrations of LLWP‐C, LLWP‐1, and LLWP‐3, that is, 1, 3, 10, 30, and 100 μg/mL, did not show any change in the cell viability for RAW264.7 murine macrophages showing little or no cytotoxic effects. All the fractions showed a positive effect on phagocytic activity of these macrophages except for 1 μg/mL of LLWP‐C polysaccharide. Both purified fractions showed potent immunostimulatory effects with the highest phagocytic activity reported by using LLWP‐3. The same fraction was also reported to enhance the secretion of NO, IL‐6, and TNF‐α, although other tested polysaccharides were also effective; however, LLWP‐3 was found to be the most efficient. Cytokine array assay also showed a positive effect of LLWP‐3 fraction on the production of 13 different cytokines. The expression of NF‐*k*B p65 and iNOS was reported to increase in experimental cells as compared to control when the three polysaccharides were used at a concentration of 10, 30, and 100 μg/mL, with LLWP‐3 being the most effective. The same fraction was also observed to have a positive effect on MAPK signaling pathway as compared to other two polysaccharides. The study suggested that besides lotus leaf polysaccharides extracted by hot water extraction, the purified fraction LLWP‐3 can be used as a powerful agent having immunostimulatory effects (Song et al., 2019).

#### 5.1.3 Antioxidant activity

Lotus leaf polysaccharides were explored for antioxidant potential by the analysis of different parameters (Zhang et al., 2015). Two polysaccharides were extracted in this study using dynamic high‐pressure microfluidization‐assisted extraction (DHPMAE), LLPs‐D, and leaching, LLPs‐L. Both were further purified; their monosaccharide composition was determined besides the elucidation of molecular weight. DPPH free radical scavenging activity showed that both polysaccharides showed the activity when tested in a concentration ranging from 0 to 1.0 mg/mL. LLPs‐D was found to exhibit better free radical scavenging activity as compared to LLPs‐L. 0.38 mg/mL concentration was required as IC_50_ value as compared to 0.99 mg/mL for LLPs‐L, showing that extraction of lotus leaf polysaccharide through DHPM potentially exhibits better antioxidant potential as compared to extraction through leaching. For hydroxyl radicals, the scavenging activity determined by the use LLPs‐D was 67.84%, and for LLPs‐L, it was 53.28% at a concentration of 1.0 mg/mL. An IC_50_ value of 0.61 mg/mL was reported for LLPs‐D and 0.92 mg/mL for LLPs‐L. Reducing power measured for both polysaccharides showed that LLPs‐D at concentration range of 0.2–1.0 mg/mL showed better reducing power as compared to LLPs‐L in a dose‐dependent manner. Reducing power for LLPs‐L was reported as 0.355 and for LLPs‐D was reported as 0.503. It was reported that LLPs‐D had less average molecular weight as compared to LLPs‐L, and both differed with respect to molar percentage of monosaccharide composition. LLPs‐D showed high percentage of rhamnose and mannose while low percentage of glucose and arabinose, and for LLPs‐L, the composition was vice versa. Shorter extraction time and potential antioxidant activity reported LLPs‐D as a prospective future candidate for therapeutic purposes (Zhang et al., 2015).

#### 5.1.4 Anti‐osteoporotic activity

Osteoporosis is an important disease that leads to bone loss and is also called a silent disease due to its stealthy progression. Usually, the disease becomes evident after its progression (Sambrook & Cooper, 2006). Besides several other factors, hormonal imbalance is an important factor leading to this condition (Weitzmann & Pacifici, 2006). Several therapies have been used for the treatment of osteoporosis including hormones therapy, but various side effects have been reported through the use of all these techniques, especially hormone therapy (Reid, 2015; Vandenbroucke et al., 2017). Natural plant‐derived compounds like polysaccharides may show potential for treating osteoporosis, and lotus leaf polysaccharide has been explored for this purpose through in vivo study (Hwang et al., 2020). The same study (Hwang et al., 2020) used female C57BL/6 mice where ovariectomy was performed for experimental group. For lotus leaf polysaccharide extracted by pectinase, LLEP was administered in two amounts LLEP‐L (low dose, 30 mg/kg/day) and LLEP‐H (high dose, 100 mg/kg/day) as a single dose per day for 4 weeks. At the end of experiment, blood, femoral, and gonadal fat samples were collected from the mice for further analysis. It was observed that LLEP inhibited the effect of obesity by successfully preventing fat accumulation. Micro‐CT analysis of the trabecular region of the distal femurs showed thick and compact trabeculae in the mice treated with LLEP, which gave morphologic evidence of positive results in terms of bone maintenance in mice treated with LLEP. The results were consistent with the trabecular thickness (Tb.Th), bone volume (BV/TV), and trabecular numbers (Tb.N). Analysis of markers that reveal the process of bone turnover was performed. C‐terminal telopeptide of type I collagen (CTX‐I), a marker for the resorption of bones, and N‐terminal propeptide of type I procollagen (PINP), a marker for bone formation, were analyzed in this study. CTX‐1 and PINP showed that untreated mice had reduced serum levels of CTX‐1 and no effect was observed on the level of PINP, while decreased level of PINP was reported in LLEP‐treated mice. The effect of treatment on osteoclast differentiation determined through tartrate‐resistant acid phosphatase (TRAP) assay showed that LLEP caused a dose‐dependent reduction (3.13–200 μg/mL) in the TRAP activity; moreover, CCK‐8 assay determined the absence of any cytotoxic effects caused by the LLEP. A downregulation of c‐Fos/NFATc1 was caused by the LLEP. All the parameters observed in this study showed positive effect against osteoporosis in experimental mice (Hwang et al., 2020). To our knowledge, this is the only report available regarding positive effect of polysaccharides from lotus plants for the prevention of osteoporosis.

### 5.2 Biological activities from polysaccharides of lotus root residues

A detail of useful biological activities exhibited by the lotus root residues is given in this section.

#### 5.2.1 Immunomodulatory activity

A water‐soluble polysaccharide (LRP) extracted from the root residues of *N. nucifera* was tested for immunomodulatory activity by Hu et al. (2019). Analysis showed that LRP was a neutral polysaccharide having 70.08% carbohydrate content, 0.16% protein, and 0.46% uronic acid. An in vitro immunomodulatory effect of LRP was determined using RAW264.7 murine macrophages. MTT assay conducted to see the effect of cytotoxicity of LRP on RAW264.7 macrophages after using it in a concentration of 12.5 to 200 μg/mL showed that LRP showed no cytotoxic effect till 100 μg/mL; however, subsequent decrease in cell viability was observed when concentrations exceeding 100 μg/mL were applied. An increase in the production of NO, TNF‐α, and IL‐6 was observed when LRP was used in a dose‐dependent manner. Maximum production of NO was recorded as 35.93 μM at 100 μg/mL without any cytotoxicity; similarly, 26.32‐fold increase in TNF‐α production and 59.18‐fold increase in IL‐6 production were observed at 100 μg/mL LRP concentration. The presence or interference of endotoxins was also tested, and the absence of endotoxins was reported, stamping the positive activity of LRP on the above‐mentioned immune system regulators. Morphological changes occurring in experimental cells using Scanning Electron Microscopy (SEM) also endorsed the above‐mentioned results. Further positive regulation of MAPK and P13K/Akt signaling pathways was also reported using the LRP. In vivo study conducted on Balb/c mice treated with 20, 40, and 80 mg/kg/day doses of LRP showed a strong stimulation of the production of inflammatory cytokines (Hu et al., 2019).

Yi et al. (2018) determined the immunostimulatory activity of polysaccharides extracted from lotus roots (LRPs). Evaluation of production of NO showed that LRPs increased the production of NO in a dose‐dependent manner. At 400 μg/mL, the production of NO was recorded to increase to 39.43 μmol/L from 18.54 μmol/L. Similarly, a positive effect on the secretion of TNF‐α was reported using LRPs. At 400 μg/mL concentration of LRPs, the production of TNF‐α increased to 303.19 pg/mL from 267.27 pg/mL. The results of this study endorsed positive immunostimulatory effect of LRP (Yi et al., 2018).

Yi et al. (2019) determined the effect of using LRPs on macrophage activation, a key regulator of the immune system by evaluating the level of production of TNF‐α and NO. A concentration range of LRPs from 0.05 to 0.80 mg/mL was tested. It was reported that secretion of NO and TNF‐α both enhanced in a dose‐dependent manner. However, concentration of LRPs in the range of 0.4–0.80 mg/mL did not show significant differences among themselves. Therefore, it was concluded that the use of both concentrations can have positive immunostimulatory effects. In case of the TNF‐α, the increase in secretions was observed to be close to control (5 μg/mL, LPS); however, the corresponding secretions in case of TNF‐α were lower. Overall, this study showed a positive effect of LRPs for showing immunostimulatory activities (Yi et al., 2019).

#### 5.2.2 Antioxidant activity

Yi et al. (2018) evaluated antioxidant potential isolated from flesh, nodes, and peels from 13 different lotus root varieties. Total of 39 polysaccharides (LRPs) were explored for their antioxidant potential by evaluating the free hydroxyl ion scavenging, DPPH radical scavenging, and FRAP assay. IC_50_ values as determined by this study ranged from 0.14 to 3.44 mg/mL for DPPH radical scavenging activity, and 0.37 to 1.27 mg/mL for hydroxyl ion scavenging activity. The FRAP activity ranged from 0.10 to 1.61 mmol/g. Generally, highest antioxidant activities were concluded to be exhibited by the node LRPs followed by the peel LRPs and flesh LRPs as determined by the FRAP assay and DPPH radical scavenging activities. However, hydroxyl radical scavenging activity concluded the highest antioxidant potential shown by the flesh LRPs, followed by both peel and node LRPs which showed approximately similar results (Yi et al., 2018).

In another study, Yi et al. (2019) estimated the antioxidant potential of lotus root polysaccharides (LRPs) by using DPPH free radical scavenging and hydroxyl radical scavenging potential through in vitro assays. 0.2–1.0 mg/mL concentration of polysaccharides was tested for establishing the hydroxyl free radical scavenging ability and a concentration of 0.5–2.5 mg/mL was tested for the DPPH free radical scavenging ability. IC_50_ value for DPPH free radicals was determined 1.65 mg/mL and IC_50_ value determined for hydroxyl radicals was 0.55 mg/mL. The amount of polysaccharide required for scavenging 1 mmol DPPH free radical was determined as 2.36 g/mmol. According to this study, the free radical scavenging ability exhibited by the LRPs was stronger as compared to polysaccharides from other plants (Yi et al., 2019).

In a recent study, Nawaz et al. (2021) determined the antioxidant potential of alkali‐soluble polysaccharide obtained from lotus rhizomes. Different parameters used for in vitro determination included evaluation of free radical scavenging potential against hydroxyl (OH) radicals, DPPH free radical scavenging activity, ferric ion reducing power (FRP), and Trolox equivalent total antioxidant activity (TAOA) for native and microwave‐treated alkali‐soluble cell wall polysaccharide (CWPs). TAOA for native and microwave‐treated CWPs was recorded in the range 3.06 ± 0.02% to 5.54 ± 0.01%; for FRP, 0.36 ± 0.005% to 0.50 ± 0.006%; for DPPH free radical scavenging activity, 3.57 ± 1.57% to 16.03 ± 0.032%; and for hydroxyl ion scavenging activity, it ranged from 59.39 ± 2.01% to 78.66 ± 0.36%. It was concluded that microwave treatment resulted in increased antioxidant potential and functional properties as compared to native CWPs. Moreover, the increase in time for microwave treatment resulted in a linear increase in antioxidant ability of CWPs (Nawaz et al., 2021).

#### 5.2.3 Antitumor activity

Yi et al. (2018) explored the antitumor activity of 39 polysaccharides isolated from 13 varieties of lotus root by testing against SGC7901 and HepG2 cells. Determination of cytotoxicity showed that no cytotoxic effect was observed against both the cell lines when LRPs were tested up to a concentration of 1000 μg/mL. For SGC7901 cells, the IC_25_ was reported from 102.74 to 1017.33 μg/mL. IC_25_ value for HepG2 cells was in the range of 57.30–1117.89 μg/mL. Among all the varieties tested, the strongest antitumor effect was reported using polysaccharide extracted from the peel of *Baipaozi* lotus root. The activity was determined strongest against both the tested cells. The weakest activity was reported using flesh polysaccharides from *Zoumayang* and *Elian* lotus root samples.

Yi et al. (2019) evaluated the antitumor activity of LRPs in vitro using SGC7901 and HepG2 cells for establishing the therapeutic potential of LRPs against gastric and liver cancers. A range of different concentrations of LRPs were used in this study, from 0.05 to 0.80 mg/mL. The growth of cells is inhibited in a dose‐dependent manner. The inhibition occurred logarithmically where linear relationship among the dose and inhibition of cells was observed. In case of HepG2, maximum inhibition of 36.30% of cells was achieved at a concentration of 0.80 mg/mL, and for SGC7901 cells, 44.25% cells were inhibited when 0.80 mg/mL LRPs were used in vitro (Yi et al., 2019).

### 5.3 Biological activities from polysaccharides of lotus seeds

In this section, we will review the useful biological activities as exhibited by the polysaccharides extracted from lotus seeds.

#### 5.3.1 Antitumor activity

Zheng, Wang, Zhuang, et al. (2016) studied the in vitro and in vivo antitumor potential of crude polysaccharide extracted from the lotus seeds (LSPS). MFC (mouse gastric cancer cells), H22 (mouse hepatocarcinoma cells), and HuH‐7 (human liver cancer cells) were treated with 0, 50, 100, and 200 μg/mL LSPS for 48 h in in vitro study. As compared to negative control, some antitumor potential was reported using LSPS in certain concentrations, and it was found more effective against HuH‐7 and H22 cells as compared to MFC cells. This showed that LSPS possesses certain potential for the treatment of liver cancer. For assessing the antitumor potential in vivo, mice bearing H22 tumor were treated by orally administering LSPS for 14 days. Mice were treated with low (50 mg/kg), medium 100 mg/kg, and high 200 mg/kg doses of LSPS to establish their therapeutic potential. Successful inhibition of cellular growth was observed in a dose‐dependent manner. 50 mg/kg oral dose resulted in 17.90% tumor inhibition, 100 mg/kg resulted in 39.60% inhibition, and 200 mg/kg resulted in 45.36% tumor inhibition. Moreover, other factors also showed a positive effect. No variation in the body weight for mice treated with LSPS was observed. Other positive factors included better appetite, better coat luster, activity, and low toxicity showing antitumor potential of LSPS in vivo (Zheng, Wang, Zhuang, et al., 2016).

#### 5.3.2 Immunomodulatory activity

Polysaccharides from lotus seeds were explored for immunomodulatory effects in vivo. Zheng, Wang, Zhuang, et al. (2016) treated H22 tumor‐bearing mice with different doses of LSPS (50, 100, and 200 mg/kg) over 14 days' time period. From these mice, spleen and thymus as important organs were removed, and their indexes were measured. Negative control and positive control (mouse treated with CTX, 30 mg/kg) were also analyzed and it was observed that treatment with CTX caused reduction in the spleen index by 7.92% and 10.19% in the thymus indexes. This reduction was not observed in a dose‐dependent manner and low dose of LSPS was found appropriate for reduction in thymus indexes which was hypothesized as an important immune activation step in the treatment of tumor. Other blood parameters meant white blood cells (WBC's), red blood cells (RBC's), Hemoglobin (Hb), and Platelets. Another analysis revealed that the levels of IL‐2 and TNF‐α were enhanced in the groups treated with LSPS resulting in the improvement of immune functions of tumor‐bearing mice. An increase in the superoxide dismutase (SOD) activity was reported in a dose‐dependent manner and a decrease in the malondialdehyde (MDA) levels was observed which also led to a conclusion of antitumor effects of LSPS in tumor‐bearing mice (Zheng, Wang, Zhuang, et al., 2016).

Liao et al. (2011) used polysaccharides isolated from lotus seed plumule (LPSS) for studying anti‐inflammatory effects on splenocytes isolated from female NOD/Lt, that is, non‐obese diabase mouse models and BALB/cBy/Narl (normal mice used as a control). Splenocytes from these mice were cultured at different ages. Determination of cell toxicity was done by treating splenocytes isolated from BALB/c mice at 10 weeks. Treatment with 0, 39, 78, 156, 312, 625, 1250, and 2500 μg/mL for 72 h at 37°C showed no cytotoxic effect for all concentrations except for 2500 μg/mL. For further analysis, 78, 312, and 1250 μg/mL concentrations of LPPS were used. The effect of LPPS on Th1 and Th2 cytokine secretions from the primary splenocytes from NOD and BALB/c at the mentioned three concentrations of LPPS was determined. The effect was observed on IL‐1β, IL‐2, and TNF‐β (Th1 cytokines), and IL‐4, IL‐5, IL‐6, and IL‐10 (Th2 cytokines), after 48 h of incubation with splenocytes from 15, 22, and 26 weeks aged both groups of mice. For IL‐1β, IL‐2, IL‐4, and IL‐5, a negligible and undetectable difference was reported. Increased production of cytokines TNF‐α, IL‐6, and IL‐10 was reported, where a significant increase in the production of IL‐6 and IL‐10 was reported at 78 and 312 μg/mL concentrations of LPPS for 22‐week‐old NOD mice, suggesting potential anti‐inflammatory activity (Liao et al., 2011).

In another study, Liao and Lin (2011) studied the effect of lotus plumule polysaccharide (LPPS) after its administration to non‐obese diabetic mice (NOD) having 15 weeks of age. Determination of Th1 cytokines (interferon (IFN)‐λ, IL‐1β, and IL‐2, and Th2 cytokines, IL‐4, IL‐5, IL‐6, and IL‐10 level) was determined in the splenocytes, and IL‐6, IL‐10, and TNF‐α level were determined in the liver and kidney. It was concluded that LPPS decreased the weight of enlarged spleen in experimental (NOD) mice. The expression of pro‐inflammatory cytokines IL‐6 and TNF‐α was inhibited, and decreased secretion ratio for IL‐6/IL‐10 was recorded in splenocyte cultures. In this study, evaluation of genetic expression also concluded that the pro‐ and anti‐inflammatory cytokine genes are regulated in case of liver. The study suggested that an overall positive effect on spleen and liver of NOD is observed after 15 weeks of LPPS treatment (Liao & Lin, 2011).

#### 5.3.3 Antioxidant activity

Tian et al. (2012) tested a crude polysaccharide extracted from the seeds of *N. nucifera* called LSPS and a purified fraction of this polysaccharide named LSPS‐1 for antioxidant potential. Hydroxyl ion scavenging ability tested in vitro showed a dose‐dependent antioxidant potential of both polysaccharides; however, crude extract was found effective and efficient as compared to the purified fraction. A concentration range of 0.1 to 0.5 mg/mL showed that hydroxyl radical scavenging activity increased with the increase in concentration, and for crude extract, increased activity was reported at all the points compared to the control and purified fraction. At 0.5 mg/mL, the antioxidant potential for LSPS was recorded as 89.51%, for LSPS‐1 60.14%, and the positive control 63.31%. An increased content of uronic acid, protein, and sulfuric radical present in the LSPS was hypothesized to be responsible for increased OH radical scavenging ability. Superoxide radical scavenging activity was also determined by using both polysaccharides in a concentration of 0.1–5 mg/mL along with positive control. In this test again an increased LSPS scavenging activity was reported as compared to purified fraction (LSPS‐1); however, unlike OH radical scavenging activity test, the activity remained less as compared to positive control (Tian et al., 2012).

#### 5.3.4 Prebiotic activity (facilitation of growth of probiotic bacteria)

There are several bacteria present in the digestive tract of human body which perform useful functions by secreting important amino acids, enzymes, etc.; however, many of them are sensitive to gastric secretions. Bifidobacteria are one of the important probiotic agents which produce at least 19 amino acids besides several enzymes. These are important precursors of pyrimidines, purines, and vitamins belonging to vitamin B (Ventura et al., 2009). Lu et al. (2015) used crude oligosaccharides obtained from lotus seeds called LOS, and a fraction of oligosaccharide was purified using microporous resin called P‐LOS. The effect of these oligosaccharides was compared with other carbohydrates, which were glucose (Glc), xylo‐oligosaccharides (XOS), and fructooligosaccharides (FOS) on the growth of *B. adolescentis* (a useful amino acid and enzyme producing bacteria of the digestive tract). All carbohydrates were tested in a concentration of 0.1, 0.3, 0.5, 0.7, 0.9, and 1.1 mg/mL individually by adjusting their concentration after 48 h of incubation. As compared to other carbohydrates, both LOS and P‐LOS were able to facilitate the growth rate of *B. adolescentis*, resulting in faster achievement of exponential phase, when used in a concentration up to 0.5 mg/mL. The viability of the *B. adolescentis* strain was increased using LOS and P‐LOS at low pH, that is, ranging from 1.5 to 3.0 at interval of 0.5. The study was significant in providing evidence of using lotus seed oligosaccharides for growth promotion of probiotic strains (Lu et al., 2015).

#### 5.3.5 Antidiabetic activity

Liao and Lin (2013a) determined the therapeutic potential of lotus plumule polysaccharides (LPPS) for the treatment of Type 1 diabetes. Non‐obese diabetic mice (NOD) and imprinting control region (ICR) mice, 7 ‐week‐old, were used in this study as experiment and control groups. The mice were fed with different doses of LPPS for 15 weeks. Low‐dose group was fed with 19.71 ± 0.87 g LPPS (0.025% LPSS added in AIN76 diet), medium was fed with 20.49 ± 1.39 g LPSS (0.125% LPPS in AIN76 diet), and high‐dose group received 19.40 ± 1.05 g LPPS (0.3125% LPPS in AIN76 diet) besides positive and negative control groups. Oral glucose tolerance test (OGTT) was performed at 0‐ and 7‐week time points. Fasting blood glucose levels at 7 weeks showed less blood glucose levels in the mice fed with LPPS as compared to the controls. After 15 weeks of treatment, the analysis of pancreatic islets was conducted after sacrificing the mice and it was observed that LPPS administration results in a greater islets cell number for the treated mice as compared to the untreated or control groups, suggesting a positive therapeutic effect of long‐term treatment of LPPS. Basal insulin secretion in the treated NOD mice also improved as compared to the untreated groups. Serum lipid profiles of diabetic mice also indicated therapeutic potential as increased level of high‐density lipoprotein cholesterol level and decreased levels of total cholesterol as well as low‐density lipoprotein levels were observed. Generally, therapeutic potential for the treatment of Type1 Diabetes and its complications associated with chronic diabetes was observed by LPPS administration through the observation of positive effect in controlling serum lipid levels and protective effects exerted on pancreatic islets after 15 weeks of LPPS administration (Liao & Lin, 2013a).

A recent study was conducted by Le et al. (2021) to examine the role of a purified fraction of *N. nucifera* lotus plumule (NNP‐2) in regulating glucose metabolism in insulin‐resistant HepG 2 cells, besides the role of NNP‐2 in promoting growth of *Lactobacillus* and *Bifidobacterium* was also explored. Supplementing the growth medium of *B. adolescentis* and *L. acidophilus* with 2% of NNP‐2 as a carbon source resulted in a significant increase in the number of colonies of both bacteria; however, in case of *B. adolescentis*, the growth was slightly reduced when 2% NNP‐2 was used as compared to insulin. NNP‐2 also showed inhibition of α‐glucosidase as determined through in vitro assay. A 40–200 μg/mL concentration range was tested which resulted in 91.32% inhibition at 200 μg/mL while IC_50_ was determined as 97.32 μg/mL. For determining the effect of NNP‐2 on glucose consumption, it was observed that 200 μg/mL of polysaccharide resulted in 158.8% increased uptake of glucose in insulin‐resistant HepG2 cells, while no cytotoxic effect of NNP‐2 on these cells was observed when concentration up to 400 μg/mL was tested. Therefore, it was concluded that 200 μg/mL concentration of NNP‐2 results in improving glucose consumption in insulin‐resistant HepG2 cells. An increased expression of IRs1, P13K, and Akt was reported in a dose‐dependent manner when 50–200 μg/mL concentration was used. The data concluded that modulation of IRS1/P13/Akt pathway occurs in the insulin‐resistant HepG2 cells which could result in improved insulin sensitivity (Le et al., 2021).

Among all the biological activities tested, it has been observed that immunomodulatory and antioxidant activity have been reported from leaves, roots, as well as seeds. Although all the polysaccharides exhibited immunomodulatory role, the cytotoxic effect that has not been reported in case of leaves is worth noting. In case of roots, seed cytotoxic effect has been reported. Polysaccharides from seeds showed a greater cytotoxic effect as compared to polysaccharides from roots. In general, antioxidant potential reported from the seeds of *N. nucifera* shows higher antioxidant potential compared to other parts but the concentration ranges tested from different parts differ. Therefore, for a better understanding, a rationalized concentration range will have to be tested keeping in view the cytotoxic potential of polysaccharides reported from the seeds and roots for a better understanding of antioxidant potential. Some biological activities have been explored from some parts, and not from others. For example, anti‐osteoporotic activity has been explored from the leaves only. Such activities should be explored from other parts as well for proper synchronization through future studies.

**FIGURE 5:**
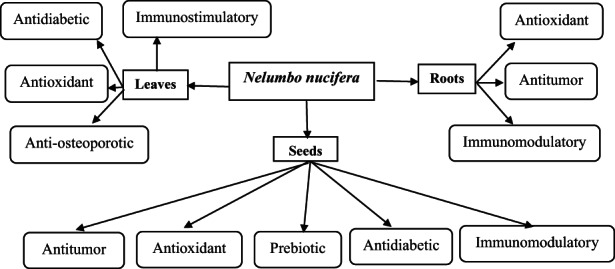
Schematic representation of biological activities from different parts of *N. nucifera*.

A schematic representation of biological activities reported from different parts of *N. nucifera* is shown in Figure 5.

## 6 CONCLUSION

*Nelumbo nucifera* is an important plant that has been used for therapeutic purposes and is an important part of human food. Leaves, roots, and seeds of this plant have been explored for extracting polysaccharides using different techniques and, in some studies, monosaccharide composition and structure have been determined. Several useful biological activities have been explored that have been discussed separately for each part of the plant in this review. These show a significant potential for this plant to be used for pharmaceutical as well as nutraceutical purposes. Antimicrobial potential has not been explored yet to the best of our knowledge from polysaccharides of this plant. Synergistic relationship of polysaccharides of this plant for therapeutic purposes remains to be studied, besides the determination of the structure of polysaccharides. Antitumor activity from the polysaccharides of leaves has not been explored yet and lotus root also possesses potential for further focus. Different extraction methods have been used for the process of extraction and it can be concluded that a combination of methods can yield better results in terms of proper polysaccharide extraction. Moreover, structure–activity relationships need to be explored further for a better understanding of the mechanisms. Temperature should be considered as an important factor and the use of membrane‐assisted separation of polysaccharides should also be applied which may achieve better results in terms of polysaccharide yield as well as potential. Therapeutic potential of *N. nucifera* elucidated in different studies mentioned in this manuscript shows that the extracts from different parts of this plant possess the ability to be used as a future therapeutic agent.

## AUTHOR CONTRIBUTIONS

**Mohib Ullah Kakar:** Writing – original draft (equal). **Hammad Karim:** Software (equal). **Ghazala Shabir:** Formal analysis (equal). **Imran Iqbal:** Methodology (equal). **Sajjad Ahmad:** Formal analysis (equal). **Muhammad Shafi:** Conceptualization (equal). **Pari Gul:** Writing – original draft (equal). **Sania Riaz:** Writing – review and editing (equal). **Rizwan‐ur‐Rehman:** Methodology (equal). **Hamid Salari:** Data curation (equal); project administration (equal).

## FUNDING INFORMATION

No funding is available for the manuscript.

## CONFLICT OF INTEREST STATEMENT

The authors declare that authors have no conflict of interest regarding publication of this manuscript.

## Data Availability

No data availability statement is required separately for the manuscript. All the data have been made available in the article.
